# Toward Elucidating the Influence of Hydrostatic Pressure Dependent Swelling Behavior in the CERCER Composite

**DOI:** 10.3390/ma16072644

**Published:** 2023-03-27

**Authors:** Jian Zhao, Zhenyue Chen, Yunmei Zhao

**Affiliations:** School of Aerospace Engineering and Applied Mechanics, Tongji University, Shanghai 200092, China

**Keywords:** CERCER composite fuel, hydrostatic pressure, multiscale simulations, fission gas swelling, finite element method

## Abstract

A ceramic–ceramic (CERCER) fuel with minor actinide-enriched ceramic fuel particles dispersed in a MgO ceramic matrix is chosen as a promising composite target for accelerator-driven systems (ADS). Fission swelling is a complex irradiation-induced phenomenon that involves recrystallization, resolution, and hydrostatic pressure under extreme conditions of high temperature and significant fission flux. In this study, a multiscale computational framework was developed to integrate simulations of continuum-scale thermo-mechanical behavior in the CERCER composite with a grain-scale hydrostatic pressure-dependent fission gas swelling model. Hydrostatic pressure-dependent fission welling is taken into account in the stress update algorithms for $UO_{2}$ particles. Accordingly, we programmed the user subroutines to define the thermo-mechanical constitutive relations in the finite element simulations. The obtained results indicate that (1) the proposed method accurately predicts the swelling deformation at various burnup levels while taking into account hydrostatic pressure and (2) prior to recrystallization, the particle swelling is primarily influenced by temperature variation, whereas after recrystallization, the presence of hydrostatic pressure favorably suppresses the swelling deformation. This work effectively captures the swelling behavior influenced by hydrostatic pressure within the dispersed-type CERCER composite fuel in ADSs.

## 1. Introduction

Accelerator-driven systems (ADS, subcritical reactors) [1,2,3] have been proposed as an efficient transmutation system to burn the long-lived radionuclides of hazardous nuclear wastes (for example, minor actinides including Np, Am, and Cm) and produce enormous amounts of energy at the same time. The main component of the reactor core is a fuel element that is loaded with MA and fissile nuclides [3,4]. The element exhibits structural similarities with rod-type fuels, which are composed of several cylindrical pellets wrapped in tubular outer cladding [5]. The pellet–cladding gap is appropriately designed to avoid mechanical interaction and the outer cladding serves as a protective shield.

The pellet used for ADS is a composite of ceramic fuel particles with MA dispersively embedded into a matrix. There are two highly preferred heterogeneous forms [3,5,6]: CERCER, which is strengthened with ceramic particles in a Mo92 metallic matrix, and CERMET, which is reinforced with the same inclusions in a MgO matrix. In previous research by the Experimental Feasibility of Targets for Transmutation (EFTTRA) group [4,7,8], uranium dioxide ($UO_{2}$) was consistently used as the particle material in CERCER targets, while MgO was an appropriate matrix choice thanks to its irradiation stability [9,10].

CERCER composites showcase sophisticated thermo-mechanical behavior when exposed to a variety of harsh conditions, such as strong, fast neurons and extreme heat, in a subcritical reactor. In addition to producing heat, the particles generate solid and gas fission products, resulting in significant irradiation swelling deformation. Post-irradiation examinations demonstrated that the swelling in $UO_{2}$ particles could reach more than 80% at deep burnups [7], and indicated that grain recrystallization occurred during this phenomenon. Recrystallization begins at the grain boundary of the original grain and progresses to the grain center until the original large grain is fragmented into small fine grains [11]. As a result, fission gas atoms can rapidly diffuse to the new grain boundary and form large intergranular bubbles. Unlike the homogeneous $UO_{2}$ pellet, the dispersed configuration restricts the release of gas bubbles, resulting in stored gas products that significantly increase material porosity, decrease thermal conductivity, and effectively scale the deformation [12,13]. Significant swelling also increases the probability of a crack by strengthening the mechanical interactions between the matrix and the fuel particles and weakening the microstructure integrity. PIE revealed radial cracking between the matrix and the particles in CERCER pellets at high burnup stages [4]. As a consequence, the evolution of in-pile thermo-mechanical behavior in the CERCER pellet should be coupled with grain-scale fission gas behavior.

In addition to experimental studies, theoretical models and numerical simulation methods should be established to explore the irradiation-induced swelling behavior in CERCER fuels to provide a comprehensive understanding of their in-pile evolution. Above all, a critical fission gas swelling model should take the grain-scale recrystallization effect into account. Theoretical models being used to describe the behavior of fission gases can be traced back to Booth’s spherical model proposed in 1957 [14]. Rest et al. [11,15] proposed a recrystallization theory for $UO_{2}$ and UMo fuels, and developed a swelling model by calculating the grain-scale fission gas diffusion behavior. Cui et al. [16] improved Rest’s model by taking hydrostatic pressure and the resolution effect of intergranular gas atoms into account, and developed a semi-analytical formula to describe fission gas behavior in UMo fuels. There have been some simulation attempts to develop efficient and precise schemes for incorporating irradiation-induced swelling behavior into various nuclear fuels [17,18,19]. Cui’s model was first employed to simulate the in-pile behavior of UMo fuel plates that were monolithic or particle-dispersed [20]. Zhang et al. [21] revised this model for calculating the effective irradiation swelling for $PuO_{2}/Zr$ inert matrices by incorporating the gas swelling model into the constitutive relations under the rotational coordinate system [17]. While computational models and numerical simulations provide insight into the thermo-mechanical behavior of various types of fuel elements, research on the three-dimensional thermo-mechanical behavior simulations of ADS fuels remains limited. Finite element analyses (FEA) [12,19,22,23], with the constructed thermo-mechanical models for $UO_{2}$ particles and MgO matrices, were performed to understand the in-pile behavior in a CERCER composite in an ADS. Ding et al. [22] used an empirical model to simulate swelling in $UO_{2}$ particles, which severely underestimated the gas swelling contribution in the CERCER pellet. In addition, the mechanistic model in [19,23] was revised from that of Cui et al. [16] to take grain-scale fission gas behavior in $UO_{2}$ particles into account. Although the authors considered intergranular gas atom resolution and investigated the evolution of intragranular swelling in a wide range of burnups, the important hydrostatic pressure was neglected. Modeling of the hydrostatic pressure-dependent swelling behavior in CERCER composites remains a significant challenge.

In this study, we aimed to improve our mechanistic understanding of the irradiation-induced swelling behavior in CERCER composites in ADS cores. The work is organized as follows. Our refined grain-scale fission gas swelling model, which includes recrystallization, gas atom resolution, and hydrostatic pressure dependence, is detailed in Section 2. Based on our previous works [12,23], we then updated the user-defined subroutines to integrate the grain-scale fission swelling model with a continuum-scale stress update algorithm and introduced finite element simulations of the multi-scale thermo-mechanical behavior of the CERCER composite pellet. We ran two simulations with and without the considered hydrostatic pressure in the gas swelling model. Section 3 and Section 4 examine the evolution of fission swelling at various burnup stages, as well as the influencing mechanism of hydrostatic pressure.

## 2. Materials and Method

### 2.1. Swelling Model with Recrystallization, Resolution and Hydrostatic Pressure

Volumetric swelling in fuel particles arises from the accumulation of fission solid products and gas fission products. Here, we adopted the volumetric engineering strain, which expresses the volumetric variation relative to its original volume:

(1) $${\left(\frac{\Delta V}{V}\right)}^{sw}=\frac{\Delta V_{\mathrm{solid}\;}}{V}+\frac{\Delta V_{\mathrm{gas}\;}}{V}$$

The solid part, $\frac{\Delta V_{\mathrm{solid}}}{V}$, grows proportionally to fission density [24,25]:

(2) $$\frac{\Delta V_{\mathrm{solid}}}{V}=2.5\times 10^{-29}\;\cdot \;F_{d}$$

$F_{d}$ (fission/m${}^{3}$) is the fission density in Equation (2), which can be calculated using:

(3) $$F_{d}=\dot{f}\times t$$

where $\dot{f}$ (fission/m${}^{3}$ s) is the fission rate and *t* (s) represents time.

The fission gas behavior that leads to considerable swelling, $\frac{\Delta V_{\mathrm{gas}}}{V}$, can be divided into two stages. Fission gas swelling is the sum of intragranular bubble swelling and intergranular bubble swelling in the first stage before recrystallization ($F_{d}\leq F_{dx}$, $F_{dx}=4\times 10^{24}\;{\left(\dot{f}\right)}^{2/15}$ [11]). The second stage occurs following recrystallization ($F_{d}>F_{dx}$), with the original grain separating into two areas. The non-recrystallized area still contains intragranular bubble swelling and intergranular bubble swelling despite the decreased grain radius. The recrystallized area with fine grains produces major intragranular bubble swelling since the gas depletion effect [26] causes fission gas atoms to migrate to the grain boundaries. Thus, the swelling of fission gas can be calculated as follows.

(4) $$\frac{\Delta V_{\mathrm{gas}}}{V}=\left\{\begin{array}{cc} {\left.\left(\frac{\Delta V_{\mathrm{intra}}}{V}\right)\right|}_{r_{gr}}+{\left.\left(\frac{\Delta V_{\mathrm{inter}}}{V}\right)\right|}_{r_{gr}} & F_{d}\leq F_{dx}\\ \left(1-V_{r}\right)\left[{\left.\left(\frac{\Delta V_{\mathrm{intra}}}{V}\right)\right|}_{r_{gr}}+{\left.\left(\frac{\Delta V_{\mathrm{inter}}}{V}\right)\right|}_{r_{gr}}\right]+{\left.V_{r}\left(\frac{\Delta V_{\mathrm{inter}}}{V}\right)\right|}_{r_{grx}} & F_{d}>F_{dx} \end{array}\right.$$

where $V_{r}$ is the volume fraction of the recrystallized area, $r_{g}r$ (m) is the current grain radius excluding the recrystallized outside, and $r_{g}rx=0.1$ μm is the fine grain radius. When the recrystallization process is completed, $V_{r}$ increases to 1.0.

According to [11], gas swelling prior to recrystallization can be expressed as:

(5) $$\frac{\Delta V_{\mathrm{gas}}}{V}=\frac{4\pi }{3}r_{b}^{3}c_{b}+\frac{2\pi R_{b}^{3}C_{b}}{r_{gr0}}$$

In Equation (5), $\frac{4\pi }{3}r_{b}^{3}c_{b}$ describes the intragranular bubble swelling, in which $r_{b}$ (n/m${}^{3}$) is the radius of the intragranular bubble and $c_{b}$ (n/m${}^{3}$) is the average concentration of intragranular bubbles. The other item, $\frac{2\pi R_{b}^{3}C_{b}}{r_{gr0}}$, expresses the intergranular bubble swelling, where $r_{gr0}$ (m) is the original grain radius, $C_{b}$ (n/m${}^{2}$) is the grain boundary bubble concentration, and $R_{b}$ (m) is the radius of the intergranular bubble. With hydrostatic pressure taken into account, the radius of the intergranular bubble, $R_{b}$, obeys the modified van der Waals gas law [11]:

(6) $$\left(\frac{2\gamma }{R_{b}}+P_{h}\right)\left(\frac{4\pi R_{b}^{3}}{3}-h_{s}b_{v}N_{b}\right)=N_{b}kT$$

where gamma (N/m) is surface tension, $P_{h}$ (Pa) is the hydrostatic pressure, *k* (J/K) is the Boltzmann constant, T (K) is the temperature, $N_{b}=N/C_{b}$ is the gas constant atom number per intergranular bubble, where N is the concentration of intergranular gas atoms, $h_{s}$ denotes the fitting parameter [24], and $b_{v}$ is the van der Waals constant for Xe. We obtained *N* by solving the governing equations [16,19] for the diffusion of fission gas atoms in the equivalent spherical grain [14], while accounting for the resolution effect in the intergranular fission gas atoms.

Equation (6) is a nonlinear equation. For a given $P_{h}$, the key $R_{b}$ can be further found via the Newton–Raphson iteration method. As a result, the correlation between $P_{h}$ and $R_{b}$ can be revised as follows:

(7) $$\frac{\partial R_{b}}{\partial p_{h}}=\frac{h_{s}b_{v}N_{b}R_{b}^{2}-\frac{4\pi }{3}R_{b}^{5}}{\frac{16\pi }{3}\gamma R_{b}^{3}+4\pi R_{b}^{4}p_{h}+2\gamma h_{s}b_{v}N_{b}}$$

As stated in the following section, the solution of hydrostatic pressure is dependent on the stress calculation.

The volumetric gas swelling in the recrystallized region is obtained as:

(8) $${\left.\left(\frac{\Delta V_{\mathrm{inter}}}{V}\right)\right|}_{r_{gx}}=\frac{4\pi R_{bx}^{3}}{3}\left(\frac{3C_{bx}}{2r_{grx}}+\frac{1}{8r_{grx}^{3}}\right)$$

where $C_{bx}$ denotes the intergranular bubble density and $R_{bx}$ is the radius of the recrystallized intergranular bubble area, which also satisfies the modified van der Waals gas law in Equation (6), where $N_{b}$ and $R_{b}$ should be replaced by $N_{bx}$ and $R_{bx}$, respectively. Similar to $R_{b}$ in Equation (7), we can obtain $\frac{\partial R_{bx}}{\partial p_{h}}$, which is expressed as:

(9) $$\frac{\partial R_{bx}}{\partial p_{h}}=\frac{h_{s}b_{v}N_{b}R_{bx}^{2}-\frac{4\pi }{3}R_{bx}^{5}}{\frac{16\pi }{3}\gamma R_{bx}^{3}+4\pi R_{bx}^{4}p_{h}+2\gamma h_{s}b_{v}N_{b}}$$

Thus, we can obtain all the expressions in the swelling calculation in Equation (1) based on the fission solid formulation (as in Equation (2)) and the fission gas bubble model (as in Equations (4)–(9)). As a result, the volumetric swelling strain can be expressed in logarithmic form as:

(10) $$\theta^{sw}=\ln\left(1+{\left(\frac{\Delta V}{V}\right)}^{sw}\right)$$

where ${\left(\frac{\Delta V}{V}\right)}^{sw}$ represents the volumetric swelling calculated in Equation (1).

Appendix Asummarizes the parameters for calculating intragranular and intergranular bubble swelling, and the other parameters can be found in [19,23].

### 2.2. Three-Dimensional Stress Update Algorithm

We established three-dimensional constitutive relations in an incremental scheme for the $UO_{2}$ and MgO matrices to simulate the thermo-mechanical behavior of the considered CERCER composite pellet. As it is a large deformation problem, we developed a stress update algorithm in a rotating coordinate system.

The solution process needs to be divided into many increments. The three-dimensional incremental constitutive relation is first given within a time increment (t,t+Δt). In brief, the relationship between Cauchy stresses, $\sigma_{ij}^{t}$, and elastic logarithmic strains, $\varepsilon_{ij}^{e(t)}$, for each integration point at time t can be expressed as:

(11) $$\sigma_{ij}^{t}=2G\left(T,t\right)\varepsilon_{ij}^{e(t)}+\lambda \left(T,t\right)\varepsilon_{\mathrm{kk}}^{e(t)}\delta_{ij}$$

where λ and *G* are the temperature and time-dependent Lamé coefficients.

Similarly, in response to a time increment, Δt, the Cauchy stresses, $\sigma_{ij}^{t+\Delta t}$, can be obtained using an updated T+ΔT and t+Δt in Equation (11). Thus, an incremental constitutive relationship can be obtained as:

(12) $$\begin{array}{cc} \Delta \sigma_{ij}= & \sigma_{ij}^{t+\Delta t}-\sigma_{ij}^{t}\\ = & 2G\left(T+\Delta T,t+\Delta t\right)\Delta \varepsilon_{ij}^{e}+\lambda \left(T+\Delta T,t+\Delta t\right)\Delta \varepsilon_{\mathrm{kk}}^{e}\delta_{ij}\\ \; & +2\Delta G\varepsilon_{ij}^{e(t+\Delta t)}+\Delta \lambda \varepsilon_{\mathrm{kk}}^{e(t+\Delta t)}\delta_{ij} \end{array}$$

where $\Delta \sigma_{ij}$ is the stress increment, $\Delta \varepsilon_{ij}$ is the elastic logarithmic strain increment, and ΔG and Δλ are the increments of the Lamé coefficients.

Based on the derived incremental constitutive relations, three-dimensional stress update algorithms for the fuel particles and the matrix need to be developed.

Logarithmic elastic strain increments for $UO_{2}$ particles are calculated by assuming that the total strain increments include elastic, thermal expansion, irradiation swelling, and plastic strain increments.

(13) $$\Delta \varepsilon_{ij}^{e}=\Delta \varepsilon_{ij}^{\mathrm{total}\;}-\Delta \varepsilon_{ij}^{th}-\Delta \varepsilon_{ij}^{sw}-\Delta \varepsilon_{ij}^{p}$$

where $\Delta \varepsilon_{ij}$ is the total strain increment and $\varepsilon_{ij}^{th}$, $\varepsilon_{ij}^{sw}$, and $\varepsilon_{ij}^{p}$ are the logarithmic strain increments of thermal, swelling, and plastic, respectively.

In particular, we specify the logarithmic strain increment of swelling as in the theoretical fission swelling model in Equation (10):

(14) $$\Delta \varepsilon_{ij}^{sw}=\frac{\theta^{sw(t+\Delta t)}}{3}\delta_{ij}-\varepsilon_{ij}^{sw(t)}$$

where $\theta^{sw}$ relates to grain-scale swelling model in Equation (10) and $\varepsilon_{ij}^{sw(t)}$ is the irradiation swelling logarithmic strains at time *t*.

The calculations for the plastic strain and thermal expansion strain can be found in [19,23]. Thus, at time t+Δt, the Cauchy stress, $\sigma_{ij}^{t+\Delta t}$, can be expressed as:

(15) $$\begin{array}{cc} \sigma_{ij}^{t+\Delta t}= & \sigma_{ij}^{t}+2G\Delta \varepsilon_{ij}^{e}+\lambda \Delta \varepsilon_{\mathrm{kk}}^{e}\delta_{ij}\\ = & \sigma_{ij}^{t}+2G\left(\Delta \varepsilon_{ij}^{\mathrm{total}}-\Delta \varepsilon_{ij}^{th}-\Delta \varepsilon_{ij}^{p}-\Delta \varepsilon_{ij}^{sw}\right)\\ \; & +\lambda \left(\Delta \varepsilon_{\mathrm{kk}}^{\mathrm{total}}-\Delta \varepsilon_{\mathrm{kk}}^{th}-\Delta \varepsilon_{\mathrm{kk}}^{sw}\right)\delta_{ij} \end{array}$$

The Cauchy stress function, $\sigma_{ij}^{t+\Delta t}$, is known to have one spherical stress component and one deviatoric stress component. In Equation (15), the swelling strains are coupled with the unknown spherical stresses [20], which have the following expression:

(16) $$\begin{array}{cc} -\frac{\sigma_{\mathrm{kk}}^{t+\Delta t}}{3}= & -\frac{\sigma_{\mathrm{kk}}^{t}}{3}-\frac{2G}{3}\left(\Delta \varepsilon_{\mathrm{kk}}^{\mathrm{total}}-\Delta \varepsilon_{\mathrm{kk}}^{th}-\Delta \varepsilon_{\mathrm{kk}}^{sw}\right)\\ \; & -\lambda \left(\Delta \varepsilon_{\mathrm{kk}}^{\mathrm{total}}-\Delta \varepsilon_{\mathrm{kk}}^{th}-\Delta \varepsilon_{\mathrm{kk}}^{sw}\right)\\ = & -\frac{\sigma_{\mathrm{kk}}^{t}}{3}-\left(\frac{2G}{3}+\lambda \right)\left(\Delta \varepsilon_{\mathrm{kk}}^{\mathrm{total}}-\Delta \varepsilon_{\mathrm{kk}}^{th}-\Delta \varepsilon_{\mathrm{kk}}^{sw}\right)\\ = & -\frac{\sigma_{\mathrm{kk}}^{t}}{3}-K\left(\Delta \varepsilon_{\mathrm{kk}}^{\mathrm{total}\;}-\Delta \varepsilon_{\mathrm{kk}}^{th}-\Delta \varepsilon_{\mathrm{kk}}^{sw}\right) \end{array}$$

in which $K=\frac{2G}{3}+\lambda$ describes the bulk modulus and the hydrostatic pressure can be calculated as $p_{h}=-\frac{\sigma_{\mathrm{kk}}^{t+\Delta t}}{3}$.

Thus, by solving the above nonlinear equation in conjunction with Equations (7) and (9), the hydrostatic pressure, $p_{h}$, can be calculated. Nonlinear iterations can be used to calculate the hydrostatic pressure, $p_{h}$, and the volumetric swelling $\theta^{sw}$ using the Newton–Raphson iteration method.

As for the MgO matrix, we consider the thermal expansion and irradiation creep effects. The thermomechanical parameters and models, including equations for calculating swelling, hardening, thermal expansion, and irradiation creep for $UO_{2}$ particles, as well as models of thermal expansion and irradiation creep in MgO matrices, have been discussed in our published works, see, for example, [12,23]. In Appendix A, we summarize the parameters used to calculate swelling. We programmed user-defined subroutines of UMAT and UMATHT to define the mechanical constitutive relations and then introduced them into the finite element simulations in ABAQUS.

### 2.3. Finite Element Model

**Figure 1:**
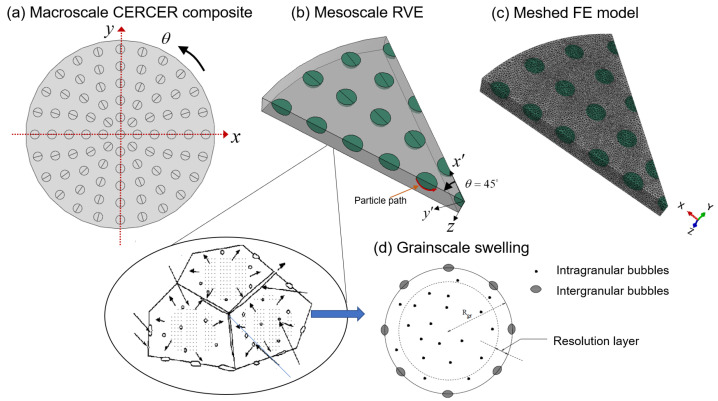
Finite element model of a CERCER composite pellet: (**a**) diagram of a CERCER composite with a heterogeneous configuration of periodically distributed fuel particles; (**b**) the RVE model; (**c**) meshing geometry in ABAQUS; and (**d**) grain-scale fission gas swelling with a spherical grain illustration showing the recrystallization process [14].

Figure 1a depicts a cylindrical-shaped model for a two-phase particulate CERCER composite with perfect particle–matrix bounding. The heterogeneous configuration was based on the assumption that the spherical particles are periodically arranged in the matrix in the axial and circumferential directions. Given the periodicity and symmetry of 1/8 in Figure 1a, we established the corresponding RVE model in Figure 1b. Therein, *x* and *y* are in-plane coordinates and *z* is the thickness. Based on the experimental reports from [4] and simulations from [12,23], we generated the geometric dimensions, with the radius of the pellet as 4.15 cm, the radius of the particle as 200 μm, and the thickness of RVE as 200 μm. The volumetric fraction of the fuel particles is 6.23%.

Given the thermo-mechanical influence of the pellet–cladding gap, we imposed a 10 MPa pressure load and a constant temperature of 873 K on the composite’s outer surface. We further applied symmetric boundary conditions on the RVE’s other surfaces, since the main deformation occurs along the radial direction of the pellet in the ADS fuel rod. Table 1 lists the loading conditions used for FE simulations, including the fission rate, the fast neutron flux, and the heat generation rate of fuel particles. For $UO_{2}$ fuel, the following formula depicts the linear relationship between the rate of heat generation and the rate of fission:

(17) $$q=c\;\cdot \;\dot{f}$$

where *q* (W/mm${}^{3}$) is the rate of heat generation and $\dot{f}$ (fission/m${}^{3}$ s) is the fission rate, and $c=3.204\times 10^{-11}\;$ (J/fission) describes the generated heat energy for every fission event.

### 2.4. Simulations and Data Analyses

We meshed the geometry using tetrahedron elements, known as C3D10MT in ABAQUS, and verified the solution convergence with a preliminary study on the density of the mesh.

The purpose of this research was to look into the effects of hydrostatic pressure-dependent fission swelling behavior in CERCER composites. Using pre-programmed UMAT and UMATHT subroutines, we ran two simulations based on the RVE model, one without the hydrostatic pressure and the other with it. In addition, we used a time unit of days to quantify the swelling evolution at various burnup stages.

## 3. Results

User-defined subroutines integrate the calculation of grain-scale irradiation-induced swelling into the continuum-scale stress update algorithm. The intergranular bubble radius, as well as the swelling contributions from intergranular and intragranular bubbles, can be captured for an integration point in the FE model, and the multiscale irradiation-induced in-pile behavior can be explored simultaneously.

We first verified the hydrostatic pressure-dependent fission swelling model that was programmed with the UMAT subroutines in this section. Then, we investigated the behavior of fission swelling and the effects of hydrostatic pressure on the in-pile behavior of the CERCER composite.

### 3.1. Model Verification

**Figure 2:**
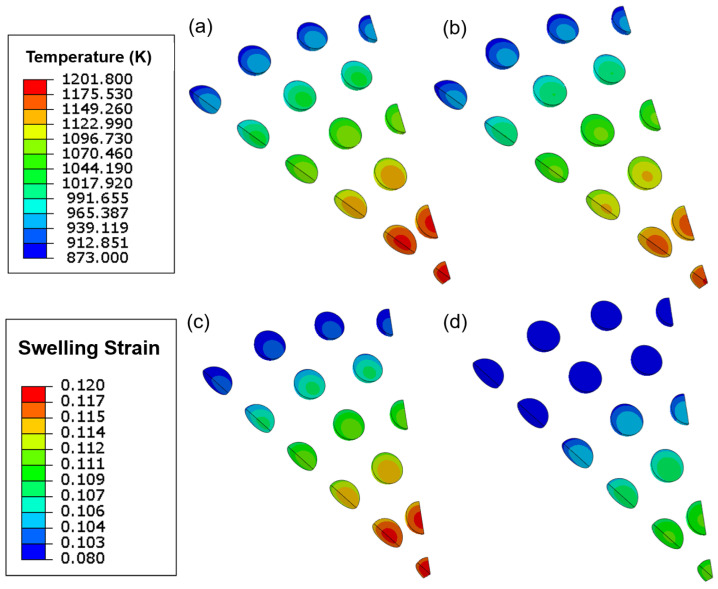
Temperature and swelling distribution contour plots on the 230th day of burnup: (**a**) temperature without hydrostatic pressure; (**b**) temperature with hydrostatic pressure; and (**c**) swelling without hydrostatic pressure; (**d**) swelling with hydrostatic pressure.

A finite element simulation of the multi-scale thermo-mechanical behavior evolution has been successfully performed. Figure 2 shows contour plots of temperature fields and the swelling strain distributions within particles on the 230th day of burnup, with comparisons between the case with and without hydrostatic pressure. The results imply that the maximum magnitudes of temperature and swelling strain are found in the center position, and that the considered hydrostatic pressure significantly reduces those values.

Since there are few experimental results for swelling descriptions at different burnups for ADS fuels, we conducted a verification of the user-defined subroutines by comparing the numerical results with the relative theoretical ones in order to validate the correct definition of the material performances.

**Figure 3:**
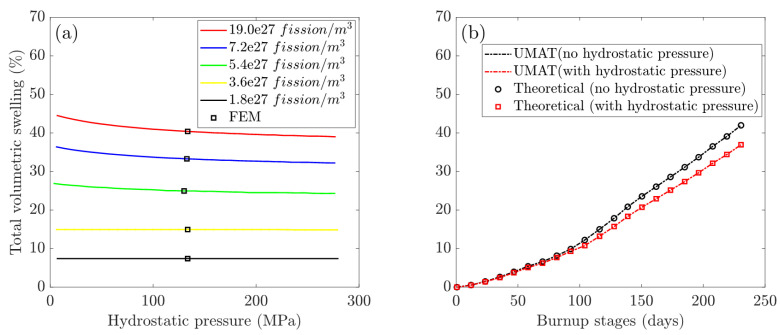
Model verification of cases with and without hydrostatic pressure: (**a**) plots of volumetric swelling vs. hydrostatic pressure with FEM results (hollow dots) compared to theoretical calculations (solid lines); (**b**) plots of volumetric swelling vs. burnup stages comparing UMAT results (dashed line) and theoretical calculations (hollow dots).

The maximum swelling strains, $\varepsilon^{sw}$, were calculated by Equation (14) from the FE model’s central integration point (cf. Figure 1). A comparison of the FEM results (hollow dots) with theoretical calculations (solid lines) of volumetric swelling at different levels of hydrostatic pressure is shown in Figure 3a. In Figure 3b, the plots of volumetric swelling vs. burnup stages compare UMAT results (dashed line) and theoretical calculations (hollow dots).

### 3.2. The Swelling Behavior of Fission within the CERCER

**Figure 4:**
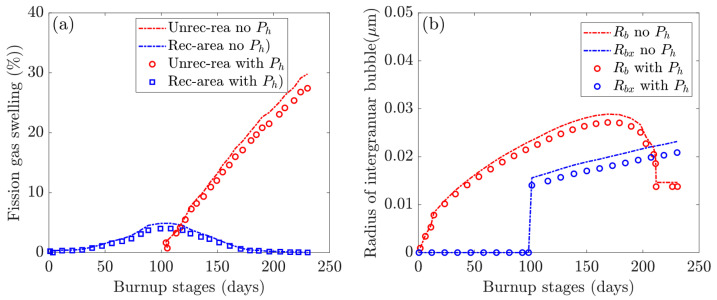
Investigations of multiscale fission swelling behavior in different simulation scenarios: (**a**) fission gas swelling in the non-recrystallized and recrystallized regions; (**b**) intergranular bubble radius in the non-recrystallized ($R_{b}$) and recrystallized ($R_{bx}$) regions.

Figure 4 depicts the multiscale swelling behavior of fission. Figure 4a depicts the evolution of fission gas swelling under hydrostatic pressure in non-recrystallized and recrystallized regions. The grain-scale radius of the intergranular bubble in non-recrystallized ($R_{b}$) and recrystallized ($R_{bx}$) regions with and without hydrostatic pressure is investigated further in Figure 4b. We extracted values at various burnup stages using the selected integration point at the pellet center (see Figure 1).

### 3.3. The Effects of Hydrostatic Pressure on the In-Pile Behavior of CERCER Composites

**Figure 5:**
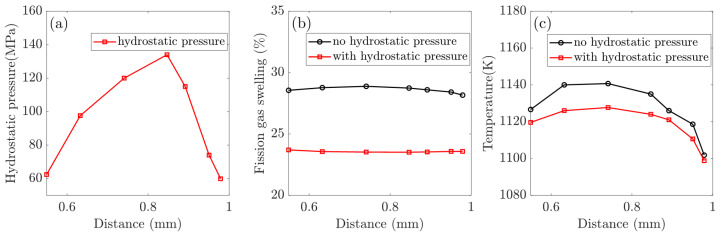
Influences of the hydrostatic pressure: (**a**) calculated hydrostatic pressure distribution along the particle path; (**b**) temperature evolution; and (**c**) fission gas swelling evolution.

**Figure 6:**
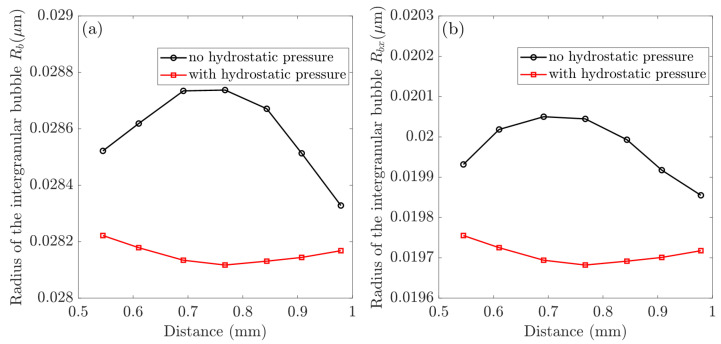
Hydrostatic pressure influences: (**a**) calculated the intergranular bubble radius in the non-recrystallized region as $R_{b}$ and (**b**) intergranular bubble radius of $R_{bx}$ in recrystallized regions.

Using selected nodes along the particle path, we investigated the effect of hydrostatic pressure on the in-pile behavior of CERCER composites. Figure 5 demonstrates the distributions of hydrostatic pressure, volumetric swelling, and temperature along the chosen path. The plots in Figure 5b,c compare the volumetric swelling and temperature variations in the gas swelling model in response to the considered hydrostatic pressure. The intergranular bubble radius in non-recrystallized and recrystallized regions is well documented to be the primary determinant of gas swelling [19], and Figure 6 investigates the changes in the bubble radius of $R_{b}$ and $R_{bx}$ in different simulation cases.

## 4. Discussion and Outlook

The numerical results of volumetric swelling agree well with the theoretical predictions, as shown in Figure 2. As a result, it can be concluded that the hydrostatic pressure-dependent gas swelling model has been successfully incorporated into the modeling framework’s continuum-scale stress update algorithm.

Irradiation-induced volumetric swelling consists of fission solid swelling and fission gas swelling, with fission gas swelling caused by gas accumulation in both recrystallized and non-recrystallized regions. Our findings show that hydrostatic pressure is important in modulating gas swelling, and its effect becomes significant at high burnup stages.

In addition, using simulations, we predicted the peak value of total volumetric swelling (on the 230th day of burnup, around 17.4% FIMA) as 43.15% (no hydrostatic pressure) and 37.14% (with hydrostatic pressure), which falls within the range of post-irradiation examination reports (47 pm, 17%) in [4]. As a result, the developed hydrostatic pressure-dependent gas swelling model can be used to describe swelling deformation in the CERCER composite pellet, and the user-defined subroutines demonstrate good accuracy and effectiveness.

### 4.1. The Swelling Behavior of Fission with the CERCER Composite

Fission swelling plots show two stages: slow swelling before recrystallization and rapid swelling kinetics during recrystallization. Fission solid swelling increases proportionally woth irradiation time, as shown by the plots in Figure 3b, and it contributes comparably to gas swelling prior to the critical fission density.

Recrystallization begins on the 97.2nd day of burnup, when the fission density reaches $2.1\times 10^{27}$ fission/m${}^{3}$. The plots show that gas swelling from recrystallized fine grains increases rapidly over time and becomes the dominant factor at high burnup stages. Furthermore, recrystallization is complete on the 218th day of burnup, which means that the original grain transforms into fine grains ($V_{r}$ in Equation (4) reduces to zero), and the total gas swelling in the non-recrystallized region vanishes.

The dominant intergranular bubble swelling in the non-recrystallized and recrystallized regions is strongly related to the intergranular bubble radius, as detailed and discussed in [19]. The generated fission gas atoms diffuse to the boundaries of the fine grains after recrystallization, resulting in accelerated gas swelling. Meanwhile, the gas swelling in the non-recrystallized region reaches a maximum at the critical fission density before gradually reducing to zero over time, indicating that the original large grain has been completely consumed. Non-recrystallized gas swelling is the sum of intragranular and intergranular bubble swelling. It should be noted that intragranular swelling remains very low, while swelling in the non-recrystallized region reaches a maximum of 6.1% at the critical density.

In Figure 4, the radius of the intergranular bubble, $R_{b}$, increases to a maximum of 0.0287 μm, then gradually decreases and remains constant after recrystallization. Nonetheless, $R_{bx}$ in the recrystallized region exhibits linear growth after the critical density. The hydrostatic pressure parameter has no effect on the evolution trend of irradiation-induced fission volumetric swelling. The total volumetric swelling accelerates quickly after recrystallization begins, and it can be seen that gas swelling in the recrystallized region eventually outnumbers solid swelling. In comparison to the hydrostatic pressure case, the total swelling decreases from 43.15% to 37.14% on the 230th day, with the dominant gas swelling in the recrystallized region decreasing from 30.73% to 25.32%. The solid swelling remains 12.42% and the gas swelling in the non-recrystallized region also decreases to zero. Simultaneously, Figure 4b shows that the hydrostatic pressure reduces the radius of intergranular bubbles. The maximum values of $R_{b}$ and $R_{bx}$ fall from 0.0287 μm to 0.0264 μm and from 0.0223 μm to 0.0191 μm, respectively.

Simultaneously, it can be observed from Figure 4b that the hydrostatic pressure decreases the radius of intergranular bubbles. The maximum values of $R_{b}$ and $R_{bx}$ decrease from 0.0287 μm to 0.0264 μm and 0.0223 μm to 0.0191 μm, respectively.

### 4.2. Hydrostatic Pressure Effects on the In-Pile Behavior of the CERCER Composite

The plotsin Figure 5 are consistent with the contour plots in Figure 2, demonstrating heterogeneous distributions of temperature and swelling in the pellet. First of all, in Figure 5a the ends of the path indicate the lowest hydrostatic pressure (approximately 60 MPa), while the middle has the highest value (approximately 140 MPa). As illustrated in Figure 5b,c, an evaluated temperature effectively promotes gas swelling, whereas hydrostatic pressure has the opposite effect, reducing the amount of swelling. Furthermore, under the combined influence of hydrostatic pressure and temperature, the gas swelling in Figure 5b tends to distribute evenly (about 24.78%). The results in Figure 6 show that hydrostatic pressure has an inhibitory effect, and that the reduction in bubble radii is the main reason for the reduced fission gas swelling. Furthermore, hydrostatic pressure dramatically reduces the bubble radius, $R_{b}$, which is shown in Figure 5a, causing the maximum values to shift from the middle (without hydrostatic pressure) to the path edges (with hydrostatic pressure).

Previous research [12,19] has investigated the mutual reinforcing mechanism between the temperature field and fission swelling. These two parameters rely on and reinforce one another. On the one hand, the increased temperature encourages gas atom diffusion to form large intergranular bubbles, resulting in increased gas fission swelling in particles [12]. On the other hand, increasing swelling causes a volumetric increase in particle inclusions, which lowers the thermal conductivity of the fuel [19] and effectively favors a temperature rise in the fuel.

It can be concluded that the presence of hydrostatic pressure is beneficial for suppressing fuel swelling, especially when high hydrostatic pressure is applied. Temperature and hydrostatic pressure have competing mechanisms for modulating fuel swelling deformations and fuel performance. At deep fission density stages, the increased temperature promotes swelling deformation, resulting in enhanced mechanical interaction and large hydrostatic pressure, which conversely lowers the swelling strain.

It is worth noting that there are no significant effects of hydrostatic pressure on fission gas swelling before the start of recrystallization, whereas the impact of hydrostatic pressure becomes more apparent after the start of recrystallization. Thus, hydrostatic pressure is important in modulating gas swelling, and its influence becomes significant at high burnup stages. The hydrostatic pressure should be considered when analyzing the in-pile behavior of CERCER pellets.

### 4.3. Limitations and Outlook

In this work, we intended to elucidate the fission swelling mechanism in CERCER composite pellets used in ADSs by implementing multiscale simulations. The applied theoretical gas swelling model considers grain-scale recrystallization, resolution, and hydrostatic pressure, which have been incorporated into the continuum-scale stress-update algorithm and realized in the FE simulations with constructed RVE particle-reinforced geometries. The model successfully predicts the swelling evolution from the initial burnup to the deep burnup stages, and the predicted values are consistent with the literature. The results suggest that hydrostatic pressure reverses the development of the gas bubble grains and thus inhibits the increase in gas swelling, especially when high hydrostatic pressure is applied. The swelling deformations at the material points are significantly decreased in response to the consideration of hydrostatic pressure. While acknowledging several limitations, we emphasized the evolution of gas wells under the effects of hydrostatic pressure in this work. The theoretical model utilized in the multiscale modeling work represents a meso-scale domain in which an atom-scale dislocation took place in the material, requiring the development of advanced algorithms and modeling tools. Concerning the continuum-scale study, we constructed an RVE model using the microstructure size reported in experiments, which requires further improvements to achieve a more realistic microstructure distribution and a higher particle volume fraction. Besides that, the MgO model could be revised based on the most up-to-date reports on MgO irradiation damage effects [6,27]. To further exploit the cracking mechanism in the MgO matrix, the interface properties of the dispersive composite should be included.

## Figures and Tables

**Table 1:** For FE simulations, loading conditions such as the fission rate, fast neutron flux, and heat generation rate of fuel particles are used.

Fission Rate	Fast Neutron Flux	Heat Generation Rate
$2.5\times 10^{20}\;$ (fission/m${}^{3}$ s)	$2.5\times 10^{15}\;$ (n/cm${}^{2}$ s)	8 (W/mm${}^{3}$)

## Data Availability

The data are available from the corresponding author on reasonable request.

## Appendix A. Description of the Gas Swelling Model for $UO_{2}$ Fuels

### Appendix A.1. Governing Equations for Gas Diffusion

The grains in $UO_{2}$ particles are roughly spherical, according to Booth’s model [14], and fission gas atoms diffuse from the grain’s interior to its boundary. With regard to gas nucleation, the diffusion equation used to describe gas atoms is as follows [26]:

(A1) $$\frac{\partial c}{\partial t}=D_{g}\left(\frac{\partial^{2}c}{\partial^{2}r}+\frac{2}{r}\frac{\partial c}{\partial r}\right)+Y\dot{f}-16\pi f_{n}D_{g}r_{g}c_{g}^{2}-4\pi r_{b}D_{g}c_{g}c_{b}+bn_{b}c_{b}$$

where *c* is the concentration of the dissolved gas, $D_{g}$ is the diffusion coefficient, *Y* is the gas yield per fission, $f_{n}$ is the nucleation factor, $r_{g}$ is the radius of the single gas atom, $c_{g}$ depicts the volumetric average concentration of the dissovled gas, $c_{b}$ is the intragranular bubble density, *b* is the intragranular resolution rate, and $n_{b}$ is the gas atom number per bubble. The analytical solution of the concentration of the gas atoms in the grain boundary can be found in [23].

### Appendix A.2. Calculation of Swelling for $UO_{2}$ Fuels

The parameters used to obtain the gas swelling before and after recrystallization are summarized in Table A1 below.

**Table A1:** The following parameters were used in the gas swelling model for swelling calculations, with recrystallization, resolution effect, and hydrostatic pressure dependency taken into account.

Parameter	Value	Unit
*Y*	0.25	-
*z*	4	-
*a*	$5.47\times 10^{-10}$	-
λ	$2.0\times 10^{-8}$	m
γ	1	N/m
$D_{0}$	$1.0\times 10^{-39}$	m${}^{5}$
$r_{g}$	$2.16\times 10^{-10}$	m
$b_{v}$	$8.5\times 10^{-29}$	m${}^{3}$/atom
$h_{s}$	0.6	-
$\delta_{gb}$	$2.0\times 10^{-9}$	m
$r_{gr0}$	$7.5\times 10^{-6}$	m
$r_{grx}$	$1.0\times 10^{-5}$	m
$B_{2}$	$2.0\times 10^{-34}$	m${}^{5}$/N

Porosity is defined as the percentage of total particle area occupied by pore space. The as-fabricated porosit, y $p_{0}$, for the $UO_{2}$ particle is set to five. The porosity changes as the burnup increases due to the accumulation of fission gas bubbles. As a result, the porosity caused by gas swelling can be determined as:

(A2) $$p_{\mathrm{gas}}=\frac{\Delta V_{\mathrm{gas}}}{V}=\frac{\Delta V_{\mathrm{gas}}}{V_{0}+\Delta V}=\frac{\frac{\Delta V_{\mathrm{gas}}}{V_{0}}}{1+\frac{\Delta V}{V_{0}}}$$

(A3) $$p=p_{0}+p_{\mathrm{gas}\;}$$

where $p_{\mathrm{gas}}$ defines the porosity caused by gas swelling, $\frac{\Delta V_{\mathrm{gas}}}{V_{0}}$ represents the volumetric gas swellings in Equation (4), and $\frac{\Delta V}{V_{0}}$ is the total swelling in Equation (4).

The evolution of porosity affects the thermo-mechanical parameters of thermal conductivity and elastic modulus; detailed expressions are available in [23]. Our previous work has discussed the evolution of porosity and its effects on thermo-mechanical parameters [12,23].

## Appendix B. Irradiation-Induced Creep in MgO Matrix

The irradiation creep needs to be involved in the matrix due to the continuous attack from fast neurons and other high-energy fragments. We used the irradiation creep model, which is similar to the CVD-SiC material, represented as

(A4) $${\bar{\dot{\epsilon }}}^{cr}=c_{0}\psi \bar{\sigma }$$

where ${\bar{\dot{\epsilon }}}^{cr}$ (/s) denotes the equivalent irradiation creep strain rate; $c_{0}$ denotes the creep coefficient, which has a value of $2.1\times 10^{-27}$ (${\left(\mathrm{MPa}\;\cdot \;n\;\cdot \;{\mathrm{cm}}^{-2}\right)}^{-1}$) in the simulations, which is based on our previous work [19]; $\bar{\sigma }$ (MPa) is the equivalent Mises stress; and ψ ($n\;\cdot \;{\mathrm{cm}}^{-2}s^{-1}$) is the fast neutron flux.

The creep model is given based on the reported linear relationship between the irradiation creep and the Mises stress for ceramic materials [28]. The thermal conductivity, thermal expansion coefficient, and Yong’s modulus of matrix material equations are all taken from [28].
